# Construction of healthy aging index from two different datasets

**DOI:** 10.3389/fpubh.2023.1231779

**Published:** 2023-09-06

**Authors:** Madara Miķelsone, Ieva Reine, Signe Tomsone, Helgi Guðmundsson, Andrejs Ivanovs, Halldór S. Guðmundsson

**Affiliations:** ^1^Statistics Unit, Riga Stradiņš University, Riga, Latvia; ^2^Department of Public Health and Caring Sciences, Uppsala University, Uppsala, Sweden; ^3^Faculty of Rehabilitation, Riga Stradiņš University, Riga, Latvia; ^4^Social Science Research Institute, University of Iceland, Reykjavik, Iceland; ^5^Faculty of Social Work, University of Iceland, Reykjavik, Iceland

**Keywords:** Survey of Health, Ageing and Retirement in Europe, wellbeing, autonomy, health, activities, cognitive, Latvia

## Abstract

**Introduction:**

The aging population presents both unique challenges and opportunities for societies around the world. To develop an effective healthy aging strategy, a tool for assessing aging process is needed. Numerous attempts to quantify the aging process have been made. However, there is still a challenge in developing and choosing a good enough score that is easy to apply, has a construct of variables that are available in most nationwide surveys for comparable results, and at the same time reflects the aging process of older individuals. The purpose of this study is to present our approach to construct a comparable Healthy Aging Index (HAI).

**Materials and methods:**

In Latvia, data from Wave 8 of the Survey of Health, Aging and Retirement in Europe (SHARE), involving 420 respondents, were used. For comparative analysis, data from a HL20 study on the health and wellbeing of the older adults in Iceland, which included 1,033 respondents, were used.

**Results:**

For Latvia, 13 items were selected, and for Iceland, nine items were selected. We constructed the HAI with four similar subscales for both countries—“Autonomy,” “Health,” “Wellbeing,” and “Activities,” and an additional subscale “Cognitive” for Latvia. We found matching items in all four subscales. For the Autonomy subscale, they were related to difficulties with everyday and daily tasks. In the Health subscale, the only matching item was self-rated physical health. One item related to loneliness was found for the Wellbeing subscale and one item related to social participation for the Activities subscale.

**Discussion:**

In our study, we found evidence for the successful construction of a HAI in two different datasets. The strength of our construct lies in the use of data from one of the largest social science panel studies in Europe (SHARE). As we were able to apply the construct to the Icelandic study, we believe that items presented in our approach are available in other population-based studies as well, and, therefore, can be easily replicated by others. By examining the existing SHARE data, HAI could be used to analyze long-term changes and could provide a foundation for comparing and monitoring the evolution of aging over time as well as comparing the aging process across societies. This is required for the authorities to conduct further analyses, proposals, and action plans in support of healthy aging.

## Introduction

Throughout the development of a definition of healthy aging, different concepts were available, starting from a negative aspect until the 1960s, when aging was considered a progressive, linear decline toward death (1). Moving forward, attitudes changed to a more positive approach. In 1987, when Rowe & Kahn developed the concept of successful aging, they emphasized the role of intrinsic and extrinsic factors on an individual’s health. However, their concept received much criticism as it focused more on the absence of disease. In 1997, they expanded their definition by addressing the importance of protective factors (2, 3). In 2002, the World Health Organization (WHO) presented the “global policy guidelines for active aging” with a definition of active aging as the process of optimizing opportunities for health, participation, and security to enhance the quality of life as people age (4). Since 2015, the WHO has developed the Healthy Aging Strategy, which is a combination of previous concepts and defines healthy aging as “the process of developing and maintaining the functional ability that enables wellbeing in older age” (5).

Healthy aging concept addresses the change in how we think, feel, and act toward age and aging. The three interrelated components of healthy aging are functional ability, intrinsic capacity, and environments (5). Functional ability combines the intrinsic capacity of the individual, the environment a person lives in, and how people interact with their environment. The key concepts are the ability to meet basic needs, to learn, grow, and make decisions, to be mobile, to build and maintain relationships, and to contribute to society. Intrinsic capacity includes locomotor (physical movement), sensory (vision, hearing), vitality (energy and balance), cognition, and psychological capacity. Information on all domains of each component can help prioritize and tailor interventions to an older person’s specific needs, preferences, and goals.

To develop an effective healthy aging strategy, a tool for assessing aging process is vital. However, there is a lack of a unanimous definition and standardized metric for the evaluation of healthy aging (6–9). The HAI serves as a valuable tool for policymakers to design evidence-based policies and programs that promote healthy aging and address the unique needs of older adults. It provides a data-driven framework for decision-making and resource allocation in such areas as healthcare, social services, and community support systems.

World Health Organization baseline report on the Decade of healthy aging has summarized 25 items in measuring intrinsic capacity and 27 items in measuring functional abilities (5). Therefore, healthy aging can be measured, even nationwide, if sufficient comparable items are included. In most studies, only some of them are reported, which does not encourage comparative research.

Recent systematic review by Behr et al. (1) shows, that 13 attempts to measure the aging process already have been published. Only one of them includes all intrinsic capacity domains (10), but lacks several functional ability domains. Some of those scores are hard to apply due to complex calculations and the construction of specific items. None of them have been replicated by others. The most common factors in those attempts included general health status, functioning and disability, quality of life (QoL), emotional and psychological health, cognitive functioning, and lifestyle factors. Less often, such factors as medical information and activities of daily living (ADL)/instrumental activities of daily living (iADL) were used.

The challenge around the world lies in developing and choosing a good enough score that is easy to apply, has a construct of variables that are available in most nationwide surveys for comparable results, and at the same time reflects the aging process of older individuals.

The purpose of this study is to examine specifically whether there is a theoretical and practical basis for developing/calculating HAI in the data of two countries. The HAI that would be constructed under the basis of available data would create a discussion and position in each country, and call for an examination of factors and conditions that creates a different score on the HAI. In addition, the use of existing data and HAIs opens up development and monitoring over time.

## Methodology

### Participants

#### Latvia

Study was based on the sample of older individuals from wave 8 of the Survey of Health, Aging and Retirement in Europe (SHARE) during the period from June till August 2020.

Survey of Health, Aging and Retirement in Europe is the largest social science panel study and has set new standards in research and scientific data collection. It has a global impact since it not only covers all EU member countries in a strictly harmonized way but additionally is embedded in a network of sister studies all over the world, from the Americas to Eastern Asia (11, 12).

#### Iceland

The wellbeing of older adults in Iceland was studied through a HL20 research conducted in 2020. The study focused on Icelandic citizens who had attained retirement age, and a simple random sample of 1,800 individuals was selected from the national registry. Out of this sample, 1,033 respondents completed the survey either via computer-assisted telephone interview (42%) or computer-assisted web interview (58%). The survey was carried out from November 2020 to January 2021.

To perform comparison between both countries, the sample of Latvia was limited to respondents aged 67 and older, with participation in both—the main questionnaire and COVID-19 add-on. Sample size for Latvia consisted of 420 respondents.

### Statistical analysis

Statistical analysis was conducted using Jamovi v.2.3.21, IBM SPSS Statistics v.27 and R v.4.3.0. For comparison of the constructed HAI such descriptive statistics measures as median and quartiles were used. To test the fitting of the created construct of HAI, a confirmatory factor analysis (CFA) was used.

*p* < 0.05 was set as the significance level.

### Construct and calculations of the healthy aging index

**Figure 1:**
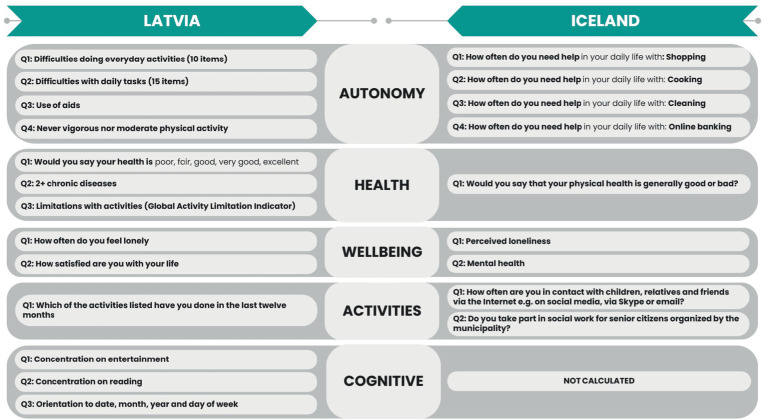
Comparison of items included in the HAI.

In the first step of constructing the HAI, we explored both datasets to find similar items for our construct (see Figure 1). For Latvia, a total of 13 items were selected. For Iceland, nine items were selected. We divided all items into five subscales. As Icelandic data did not cover the cognitive aspect of healthy aging, this subscale was used only for Latvia.

**Figure 2:**
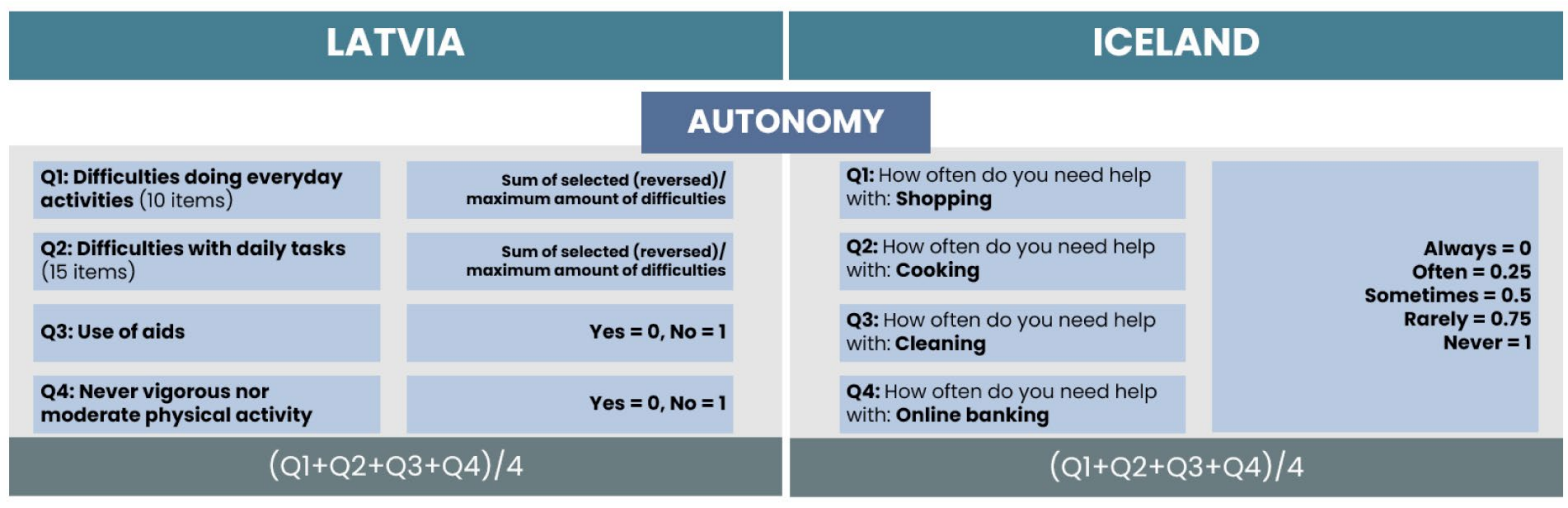
Comparison of items and calculation process for the Autonomy subscale.

**Figure 3:**
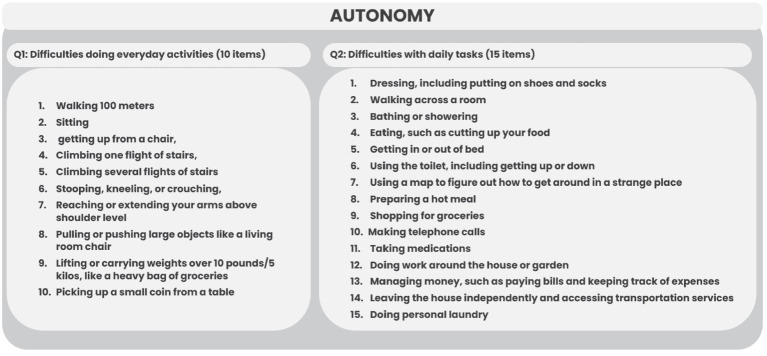
List of difficulties doing everyday activities and daily tasks for the HAI of Latvia.

For the Autonomy subscale, four items were selected for both countries (see Figure 2). We found matching items for both countries related to difficulties with everyday and daily tasks. The only difference in the statement of the question and the received responses. In Latvia, respondents were asked to select from 10 everyday tasks or 15 daily tasks with answers of “selected” or “not selected” (see Figure 3).

In the Icelandic data, four daily tasks were included, with a statement to rate the frequency of help needed. For calculation of the HAI in the Latvian dataset, we counted the total amount of selected difficulties for each respondent, reversed the score (0 difficulties as 10 points, 10 difficulties as 0 points, 0 difficulties as 15 points, and 15 difficulties as 0 points), and divided by the maximum number of difficulties reached. For Icelandic data, we coded the frequency of help needed from 0 to 1, where 0 indicates that help is always needed and 1 indicates that help is never needed. Two additional items were selected for Latvia related to the use of aids and the level of physical activity. As they were on a dichotomous scale, the coding of 0 or 1 was applied. The total score of the autonomy subscale is the average value of the scores of all items.

**Figure 4:**
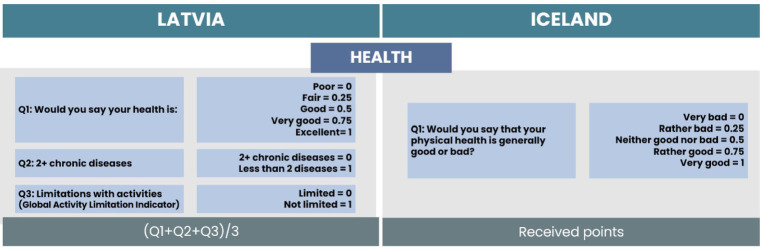
Comparison of items and calculation process for the Health subscale.

For the Health subscale, we found one matching item for both countries related to self-rated physical health (see Figure 4). The answers were on a five-point Likert type scale. From the Latvian dataset, two additional items were selected related to the presence of chronic diseases and limitations with activities (global activity limitation index). As they were on a dichotomous scale, the coding of 0 or 1 was applied. The total score of the health subscale is the average value of the scores of all items. In the Icelandic dataset, this was the only item for health, and the received points are the total score of the subscale.

**Figure 5:**
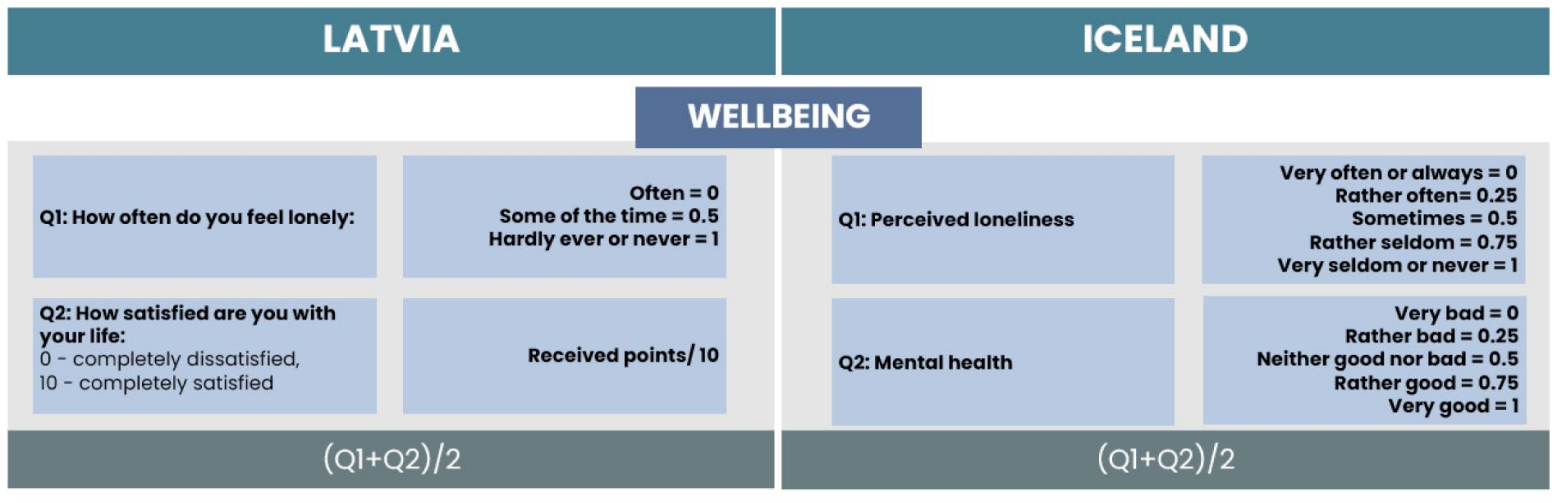
Comparison of items and calculation process for the Wellbeing subscale.

For the Wellbeing subscale, two items were selected for both countries. We found one matching item related to loneliness (see Figure 5). The only difference is in the received responses; in Latvia, respondents were asked to rate the frequency of loneliness on a three-point scale. For Iceland, the answers were on a five-point Likert type scale. The responses can be compared as “Often” can be related to “Very often or always,” “Some of the time” with “Sometimes,” and “Hardly ever or never” with “Very seldom or never” (see Figure 5). For both countries, responses were coded from 0 to 1, where 0 indicates feelings of loneliness very seldom or never/hardly ever or never and 1 indicates feelings of loneliness very often or always/often.

For Latvia, we included one additional item related to satisfaction with life. The answers were on a 10-point scale, where the received points were divided by 10. For Iceland, we included one additional item related to mental health. The answers were on a five-point Likert-type scale, and we applied a similar approach for coding the data, where 0 points indicate very bad mental health and 1 point indicates very good mental health. The total score of the wellbeing subscale is the average value of the scores of all items.

**Figure 6:**
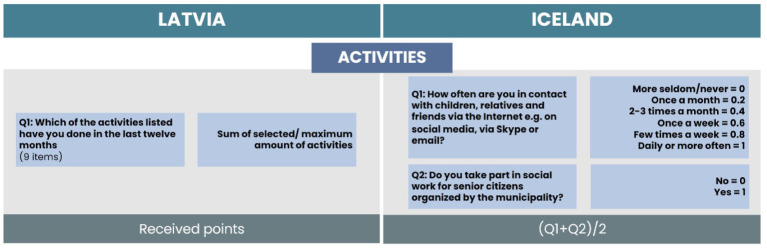
Comparison of items and calculation process for the Activities subscale.

For the Activities subscale, one item for Latvia and two items for Iceland were selected (see Figure 6). The chosen item for Latvia included nine items from different social activities. For calculation of the HAI in the Latvian dataset, we counted the total amount of selected activities for each respondent and divided it by the maximum number of activities reached. For Icelandic data, one item was related to electronic contact frequency with children, relatives, and friends. We coded the frequency of contacts from 0 to 1, where 0 indicates more seldom or never contact and 1 indicates daily or more often electronic contact frequency. The second included item for Iceland was related to taking part in social work for senior citizens organized by the municipality. As responses were dichotomous, we coded them with 0 for no such activities and 1 for participation in such activities. The total score of the activities subscale is the average value of the scores of all items.

**Figure 7:**
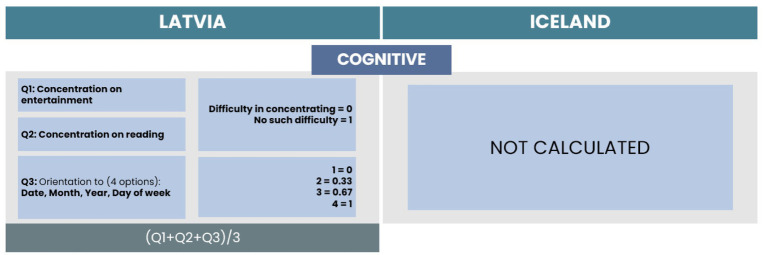
Comparison of items and calculation process for the Cognitive subscale.

We found three items related to the Cognitive subscale for Latvia (see Figure 7). For Iceland this subscale is not calculated as no items were present in a dataset. Two items were related to concentration on entertainment and reading, and one item was related to orientation to date, time, year, and day of the week. The dichotomous responses for concentration on entertainment and reading were coded with 0 and 1, where 0 indicates difficulties in concentration and 1 indicates no such difficulties. Responses regarding orientation were on a four-point scale, where 0 indicates bad orientation in each item (date, month, year, and day of the week) and 4 indicates good orientation in all four items. The total score of the cognitive subscale is the average value of the scores of all items.

**Figure 8:**
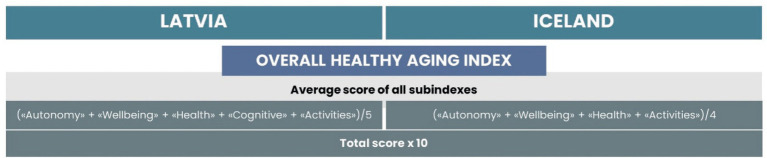
Calculation of overall HAI.

For further analysis to compare the overall process of healthy aging, we calculated the outcome as the average score of all subscales (see Figure 8). For easier interpretation, the total score was multiplied by 10.

## Results

### Factor loadings and fit indices of the constructed healthy aging index

Results for confirmatory factor analysis for both Latvia and Iceland showed high factor loadings between items and factors. For Latvia, the lowest loading was 0.23 and the highest 0.89 with most loadings being in the range of 0.47–0.89; for Iceland apart from one loading being −0.05, the lowest loading was −0.50; and the highest loading was 0.92.

Factor solution for both countries showed a good fit (see Table 1). For both the Latvian and Icelandic solution, most of the fit indexes we looked at indicated a good fit, only SRMR was slightly above the threshold for Iceland (13).

**Table 1:** Model fit for confirmatory factor analysis for the HAI.^*^

	Chi-square	Df	*p* value	Root mean square error of approximation (RMSEA)	Standardized root mean square residual (SRMR)	Non normal fit index (NNFI)	Comparative fit index (CFI)	Adjusted goodness of fit (AGFI)
Latvia	51.8	32	0.015	0.039	0.048	0.986	0.990	0.999
Iceland	104.6	32	<0.001	0.049	0.058	0.975	0.982	0.989

* As Cognitive subscale was not calculated for Iceland, it was excluded from the CFA for Latvia.

### Comparison of overall healthy aging index and its subscales

The construct of the HAI can measure physical, mental capacities (intrinsic capacity) and functional abilities of older individuals, according to the WHO conceptual model of healthy aging. The overall score of our construct is measured on a 10-point scale, where 10—indicates healthy aging (less or no decline in items related to mental, physical, and functional abilities) and 0—indicates “unhealthy” aging (more difficulties, lower levels of self-rated health and satisfaction with life, higher levels of loneliness, and less participation in social activities).

**Figure 9:**
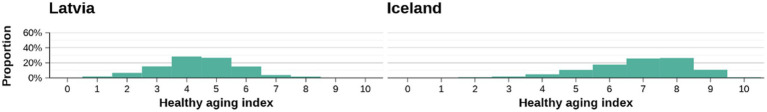
Distribution of the HAI.

Overall, the distribution of the HAI for older individuals in Latvia was lower, compared to Iceland, with median points for Latvia of 4.44 (without Cognitive subscale) and for Iceland of 7.03 points (see Figure 9). The lower score for Latvia is due to fewer points scored in the subscales of Health (self-rated health, presence of chronic diseases, and limitations with activities) and Activities (less participation in social activities).

**Figure 10:**
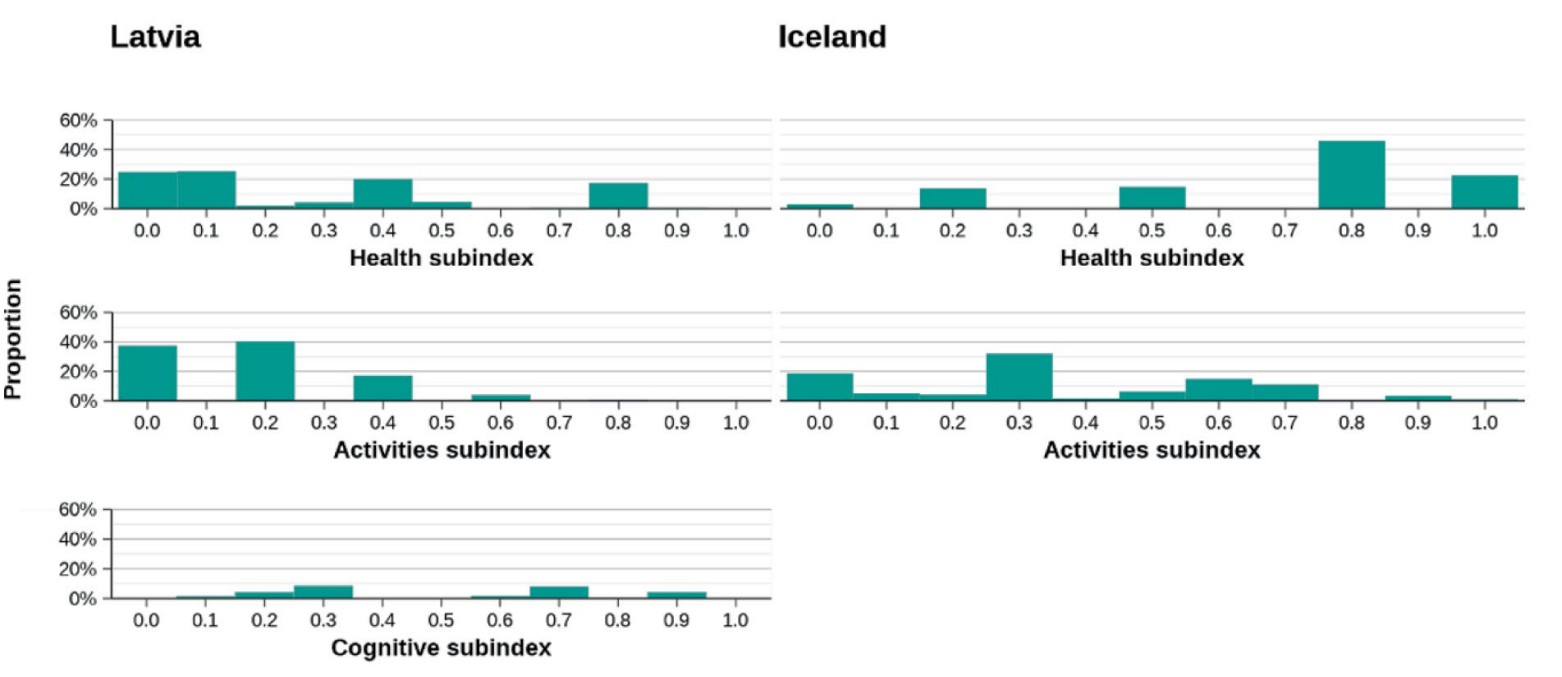
Distribution for subscales with lower scores.

The comparison of subscales for both countries shows that lower scores are obtained in such subscales as Health, Activities, and Cognitive for Latvia (see Figure 10).

**Figure 11:**
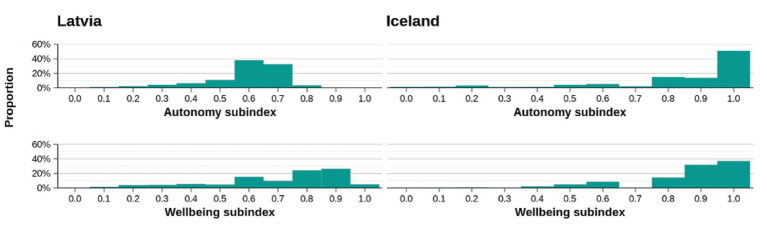
Distribution for the subscales with higher scores.

The highest obtained scores for both countries were in the subscales of Autonomy and Wellbeing (see Figure 11).

## Discussion

### On the datasets

The main goal of the study was to examine if and how the different datasets of two countries could be used to develop an index that could be useful for comparison and further discussion on the factors contributing to healthy aging in diverse cultural and societal contexts. It can also facilitate knowledge sharing and the exchange of best practices among countries.

In our study, we reached the aim as regard constructing a comparable Healthy Aging Index (HAI) from two different datasets. For Latvia, 13 items and for Iceland, nine items were used. We constructed HAI with four similar subscales for both countries—“Autonomy,” “Health,” “Wellbeing,” and “Activities,” and an additional subscale “Cognitive” for Latvia.

Furthermore, in our study, we demonstrated that items measuring underlying concepts of healthy aging can be found not only in large datasets, like the Survey of Health, Aging, and Retirement in Europe (SHARE) survey, but also in a variety of national datasets, e.g., the Icelandic HL20 study.

Although the available datasets from different population studies may not be completely identical, they might share similar items aligned with the theoretical framework. Conducting further analysis could ascertain whether these datasets form the HAI index and its subcomponents in each respective case.

The use of data from one of Europe’s largest social science panel studies (SHARE) contributes to the strength of our model. As we were able to apply the construct to the Icelandic HL20 study, the items presented in our approach can be easily found in other population-based studies, including all countries that participate in the SHARE survey, and can thus be easily replicated by others. We examined the available data for other countries participating in SHARE. The results indicate that there is a suitable foundation for comparing our HAI model across different countries. However, any additional in-depth discussion or detailed explanation of these findings falls outside the scope of this article.

Utilizing the existing secondary data offers a cost-effective, time-efficient, and flexible approach for research. It enables researchers to access large sample sizes, conduct longitudinal analyses, and perform comparative studies without the financial burden of primary data collection.

### On the method

After analyzing the available data from both countries, we conducted a theoretical review of the factors relevant to the existing and recent literature on the HAI. Subsequently, we performed confirmatory factor analysis (CFA) on both datasets and successfully confirmed the presence of HAIs in them. There are several different methods that might be used for the construction of an index, for example the UNDP approach (14, 15), Principal component analysis (16) etc. CFA was the most suitable method as it allowed us to test specific hypotheses regarding the underlying factor structure and the model’s fit to the data.

There is ongoing discussion related to the development of new healthy aging measurement tools, with the implication of focusing more on the validation of existing ones (1). Before constructing our approach, we also reviewed some of the existing scores to assess differences and similarities.

### On the theoretical approach and practical use

One of the remaining challenges is related to the lack of a unified definition of healthy aging. Rodriguez-Laso et al. (17) tested four different definitions of healthy aging. The measured prevalence of more positive results toward healthy aging increased depending on definitions, rising from 4.5% for Rowe and Kahn’s definition (freedom from disease, disability, and risk factors for disease; high physical function; and cognitive function at or above the median for one’s gender and age group) to 49.2% for a definition with fewer criteria (freedom from disability; high physical function; and freedom from severe cognitive impairment). Freedom from disease and risk factors for disease were the main reasons for not meeting the more rigorous definitions.

To reduce such bias, our construct of HAI is based on a combination of various definitions. The main factors selected for our construct are related to being free of chronic diseases, autonomy in activities of daily living, wellbeing, good quality of life, high social participation, only mild cognitive or functional impairment, and little or no disability (18). Instead of using the factor “being free of chronic diseases” due to its criticism, we chose the item “prevalence of at least two chronic diseases.”

From all existing attempts to measure the aging process, three of them used the SHARE survey (10, 19, 20). We have utilized similar approach as in a study by Sanchez-Niubo et al. (10), where they also based their theoretical framework on the WHO concept of healthy aging. The strength of their methodology lies in the use of two large studies: the Aging Trajectories of Health-Longitudinal Opportunities and Synergies (ATHLOS) project, with the data of 16 international cohorts, and the SHARE survey. It leads to the conclusion that items, related to concepts of healthy aging, can easily be found in various studies. They used item response theory (IRT) modeling, which allows testing and adjusting the effect of potential confounders on individual responses, such as the specific effects of cohorts, gender, and cultural factors. From all the attempts to measure aging process, this can be considered one of the best. However, we also found some weaknesses related to replicability issues in this approach. First, they were able to cover all domains of intrinsic capacity by including 41 items, but for replicability, all items need to be present in a dataset. By exploring the available data for Latvia from the SHARE survey, some factors were not available, and this issue might be relevant for other SHARE participant countries as well. Second, no items related to functional abilities were present. We found only one study that used the same scoring for that outcome (7).

The ideal construct of a healthy aging measurement tool also depends on standardized items within each domain. Some of the domains of intrinsic capacity were represented in almost all existing studies; however, all domains of intrinsic capacity were included in only three of them (7, 10, 21, 22). Such domains as vitality, cognition, and psychology are the most commonly used, i.e., 76% of the reviewed studies reported them. Such domains as locomotion and sensory were reported in only 4–5 studies (7, 10, 23–25).

We deliberately did not include factors related to the sensory domain, as no such items were available in the Icelandic dataset, and items regarding sensory functions have little or no impact on healthy aging, compared to other factors.

The most different approach exists in the vitality domain. Biomarkers and functional tests (Cystatin C, CRP, glomerular filtration rate, level of blood glucose, creatinine, systolic blood pressure, peak expiratory flow, and BMI) were used in some studies for measuring the vitality domain (26–31). However, the presence of chronic diseases, limitations in physical functions, and factors related to self-rated physical health are more frequently used to measure vitality.

According to the WHO, vitality domains can be measured with different types of biomarkers. At the same time, they also state that such biomarkers as body mass index, weight, waist circumference, waist-to-hip ratio, and calf circumference can lead to bias due to inaccuracy for older people due to posture, scoliosis, variability by age, sex, and ethnicity, as well as problems with selecting appropriate cut-off points (5). Inclusion of biomarkers in the construct of the HAI can strengthen the limitation of the replicability of developed scores, as specific biomarkers in large cohort studies might not be available.

However, we noticed similarities in measuring psychological domains and functional abilities. In most of the studies, items related to functional abilities—ADL or iADL and difficulties performing the activities of daily living and everyday tasks were used (17). Psychosocial factors, such as wellbeing, depression, and loneliness, are important measures, especially since the COVID-19 pandemic. As these measures are often included in health and retirement studies and are proposed by the WHO for assessment of functional abilities, they can be used in a standardized score of healthy aging (5, 10, 21, 24, 32–35). Previous research related to the exploration of the intrinsic capacity factors for the Latvian population also confirms this evidence (36, 37).

Another vital component of healthy aging is social wellbeing, which can be investigated through questions about participation in different social activities, social networks, and social support (1). They were present in some of the reviewed studies (7, 21, 22, 24, 29, 33, 35, 38). The only factor included in our construct related to social wellbeing was participation in various social activities.

### On statistics

An ideal approach for evaluating healthy aging lies in a simple score, scale, or index that weights domains of intrinsic capacity, functional ability, and environment (1). However, the universal weighing procedure is almost impossible. The importance of each domain may vary across different populations and can have significant variations in individual preferences and priorities when it comes to healthy aging. The final score of our construct is based on the average value of all subscales. The HAI composite indicator was calculated with equal weighting (EW) for each dimension. Further, each item in the dimensions had an EW. Since assigning different weights to the underlying dimensions is subjective; assigning different weights would express different importance of the items. There is no existing evidence about the importance of different items of the HAI. This approach provides a more balanced representation of each subscale by considering multiple indicators rather than relying on a single item.

There are different data preprocessing techniques for item scaling. Normalization is used to eliminate the influence of the original scale or units of measurement, and is commonly used in PCA. Min-Max normalization is useful to scale the data to a specific range and preserve the original data’s distribution and relationships, but this approach is sensitive to outliers, and extreme values may disproportionately influence the scaling. We performed a scaling procedure that is similar to the Min-Max approach: responses for each item were transformed from 0 to 1 for easier interpretation and comparison.

After scaling, the total score for each country for HAI is on a scale of 1–10. The resulting values provide a relative reflection of the variables present in the data of each country. These scores, both theoretically and statistically, form the basis for constructing the HAI.

Scores or scales also involve the danger of oversimplification. Oversimplification can happen if multiple measures are dichotomized and summed to build a quantitative score instead of using the multidimensional underlying data. Some of the reviewed studies used the percentile approach for grouping the outcome, which links the score to the characteristics of the cohort utilized and is not feasible for different cohorts (26–28, 31, 34, 39, 40). The categorization of outcomes involved statements such as “healthiest and unhealthiest,” “healthiest, less healthy, and least healthy,” “healthy aging, intermediate aging, and poor aging.”

Our approach to the construct has less bias regarding the dichotomization of items included and the outcome itself. We used original responses for items included in the construct for both countries. We chose the outcome of our construct as a score because a continuous outcome can capture a broader range of responses and provide more nuanced insights into the measured construct. Also, scores can promote greater statistical power, measurement validity, and flexibility in data analysis.

Besides the limitations related to the lack of consensus and standardization regarding the definition and assessment of healthy aging a balance between complexity and simplicity is needed. As our construct is based on different datasets, linguistic bias can also be present regarding the way a question is phrased or the specific words used, as it can prime respondents to think in a particular way or direct their attention to certain aspects. It can be reduced by employing the same methodology consistently across different populations and using standardized language and questionnaires, as well as ensuring clarity and neutrality in the wording of questions.

Validation and long-term evaluation of the developed index are critical. Assessment of its long-term predictive value can ensure that the index remains relevant and reliable in assessing healthy aging outcomes.

The diversity of the data presents both a challenge and an opportunity for exploration. The demographic variations among communities manifest in the diverse range of scores, thereby highlighting the strengths and weaknesses of each respective society. The findings of this study, along with the HAI index for each country, serve as a foundation for stimulating discussions and explanations regarding the reasons behind the observed deviations among countries. Through such discussions, insights into the disparities and underlying factors can be gained, fostering a deeper understanding of the societal dynamics at play.

In conclusion, the used approach to develop and test the Healthy Aging Index (HAI) could serve for validation of other scales to measure aging processes.

Our HAI construct has proven usefulness in examining and comparing the aging process in various societies. For example, by examining existing the SHARE data, HAI could be used to analyze long-term changes. In this way, the HAI could provide a foundation for comparing and monitoring the evolution of aging over time, as well as comparing the aging process across societies. In this way, a clearer picture of the aging process can be obtained, particularly with regard to those personal abilities that tend to deteriorate with age. This image is required for the authorities to conduct further analyses, proposals, and action plans in support of healthy aging.

## Data availability statement

The raw data supporting the conclusions of this article will be made available by the authors, without undue reservation.

## Author contributions

MM, IR, ST, and HaG: conceptualization. MM, ST, IR, HeG, and HaG: methodology. MM and HeG: software and formal analysis. AI: validation. MM: investigation, writing—original draft preparation, and visualization. IR, HaG, ST, and AI: writing—review and editing and supervision. All authors contributed to the article and approved the submitted version.

## Funding

Collaboration between Riga Stradiņš University and the University of Iceland was supported by the EEA initiative FM2021/23 (EEZ/NOFI/DIV) in the project “Modeling of the Impact of COVID-19 on Public Health of Elderly People” in Latvia and Iceland. The use of the Icelandic data from HL20 is based on the consent from all funding. The use of Icelandic HL20 data is based on the approval of public bodies and other parties that originally organized and paid for the costs of the research.

## Conflict of interest

The authors declare that the research was conducted in the absence of any commercial or financial relationships that could be construed as a potential conflict of interest.

## Publisher’s note

All claims expressed in this article are solely those of the authors and do not necessarily represent those of their affiliated organizations, or those of the publisher, the editors and the reviewers. Any product that may be evaluated in this article, or claim that may be made by its manufacturer, is not guaranteed or endorsed by the publisher.
